# Infliximab for intravenous immunoglobulin-resistant Kawasaki disease complicated with cholestatic hepatitis: a case report and discussion on coronary artery aneurysm prevention

**DOI:** 10.3389/fped.2026.1794686

**Published:** 2026-04-15

**Authors:** Zhong Yongxing, Zhang Zheng, Zheng Qi, Yang Fangyan

**Affiliations:** 1 Shaoxing Maternal and Child Health Hospital, Shaoxing, Zhejiang, China; 2 Children's Hospital of Zhejiang University School of Medicine, Hangzhou, Zhejiang, China

**Keywords:** case report, cholestatic hepatitis, coronary artery aneurysm, infliximab, IVIG resistance, kawasaki disease

## Abstract

**Background:**

Kawasaki disease (KD) is the leading cause of acquired heart disease in children under 5 years of age worldwide. Approximately 15%–20% of KD patients are resistant to intravenous immunoglobulin (IVIG), and these patients have a significantly higher risk of developing coronary artery aneurysms (CAAs), a severe complication leading to long-term cardiovascular morbidity. Cholestatic hepatitis is a rare manifestation of KD, which further increases the difficulty of clinical treatment. This study reports a case of IVIG-resistant KD complicated with cholestatic hepatitis treated with infliximab, and explores the clinical challenges in preventing CAA progression.

**Case report:**

A 2-year-old Han Chinese male presented with persistent fever as the initial symptom, followed by typical clinical manifestations of KD and cholestatic hepatitis. Initial treatment with high-dose IVIG (2 g/kg) combined with clopidogrel (1 mg/kg/day) was ineffective. A second dose of IVIG (1 g/kg) combined with high-dose methylprednisolone pulse therapy also failed to control the disease. On the ninth day of illness, salvage therapy with the tumor necrosis factor-alpha (TNF-α) inhibitor infliximab (5 mg/kg) was administered, and the child's fever subsided rapidly within 24 h. Subsequent laboratory examinations showed that inflammatory indicators [C-reactive protein (CRP), erythrocyte sedimentation rate (ESR)] and liver function indicators [alanine aminotransferase (ALT), aspartate aminotransferase (AST), bilirubin, total bile acids (TBA)] improved significantly. However, despite effective inflammation control, echocardiographic follow-up revealed progressive development of CAAs, eventually forming giant CAAs.

**Conclusion:**

Infliximab can effectively suppress systemic inflammation and improve liver function in patients with IVIG-resistant KD complicated with cholestatic hepatitis. However, the development of giant CAAs in this case underscores the dissociation between systemic inflammation control and coronary protection, highlighting the critical importance of optimizing the timing of infliximab administration. Further randomized controlled trials are needed to clarify the optimal timing, dose, and patient selection criteria for infliximab in the treatment of IVIG-resistant KD.

## 1 Introduction

Kawasaki disease (KD) is the leading cause of acquired heart disease in children under 5 years old globally, with an incidence of 5–18 per 100,000 children in Europe and the United States, and according to the 2017 AHA scientific statement, the incidence in Japan is 243.1–264.8 per 100,000 children under 5 years of age, the highest worldwide (1, 2). It is characterized by acute, self-limiting systemic vasculitis that primarily involves medium-sized arteries, with the coronary arteries being the most commonly affected target organs (1, 2). Although most cases are acute and self-limiting (1), KD-related coronary artery lesions (CALs) are associated with long-term cardiovascular risks, including early atherosclerosis, myocardial ischemia, and heart failure (3, 4).

The etiology and pathogenesis of KD remain incompletely clear. Current evidence suggests that KD results from an excessive immune response to environmental triggers in genetically susceptible individuals (4). This immune dysregulation leads to widespread endothelial dysfunction, inflammatory infiltration of the vascular wall, and activation of a pro-inflammatory cytokine network (4, 5). Recent insights propose that KD is not a direct pathogen-induced cytopathy but rather an aberrant host immune reaction to etiological substances, such as pathogenic proteins derived from infected cells, as described in the “protein homeostasis system” hypothesis (6). This framework helps explain the self-limited nature of KD and the central role of cytokine storms in vascular injury (7).

The main therapeutic goal of KD is to rapidly suppress systemic inflammation, prevent the progression of vasculitis, and minimize coronary artery damage. High-dose intravenous immunoglobulin (IVIG) combined with aspirin constitutes first-line treatment, and administration within the first 10 days of illness can reduce the incidence of CALs from approximately 25% to less than 5% (8). Nevertheless, 15% to 20% of KD patients exhibit resistant to IVIG, defined as recrudescent or persistent fever at least 36 h after the completion of the initial IVIG infusion (8). These IVIG-resistant patients face a nine-fold higher risk of developing coronary artery aneurysms (CAAs) compared to IVIG-responsive individuals (9), underscoring the critical need for timely and effective salvage therapy.

The management of IVIG-resistant KD remains a clinical challenge, with no universally accepted treatment guidelines (10). Existing salvage treatment regimens include repeated IVIG infusion, high-dose glucocorticoids, immunosuppressants (cyclosporine A, methotrexate), biological agents targeting key inflammatory mediators, and plasma exchange for refractory cases (10). Although glucocorticoids are frequently employed as second-line therapy, their use is tempered by potential drawbacks, such as delayed coronary artery healing (10). Biological agents, especially tumor necrosis factor-alpha (TNF-α) inhibitors, have emerged as promising alternative treatments due to their targeted anti-inflammatory effects.

TNF-α plays a central role in the inflammatory cascade of KD, with elevated serum TNF-α levels during the acute phase, which are even higher in patients with CALs and IVIG resistance (2, 4). Infliximab, a chimeric monoclonal antibody that specifically binds to TNF-α and inhibits its biological activity (11), has been increasingly confirmed as an effective salvage treatment for IVIG-resistant KD (12). Recent studies have shown that infliximab can rapidly resolve fever, normalize inflammatory indicators, and may promote CAA regression (1, 13). However, whether early infliximab administration can definitively prevent CAA formation, particularly in patients with extreme inflammatory phenotypes, remains incompletely established.

Hepatic involvement in Kawasaki disease is not rare; indeed, subclinical elevation occurs in 30%–50% of acute-phase patients (1, 4). Gallbladder hydrops is also a relatively frequent abdominal complication. However, overt cholestatic jaundice, with marked elevation of total and direct bilirubin and total bile acids, is a distinctly rare and severe manifestation, reported in less than 5% of KD cases (14, 15). The pathogenesis of cholestatic hepatitis complicated by KD is not yet clear; potential pathways include local inflammation caused by natural killer (NK) cell aggregation in hepatic sinusoids during the acute phase, vasculitis of small hepatic and biliary vessels affecting bile flow, and immune-mediated liver injury induced by pro-inflammatory cytokines (IL-1, IL-6, TNF-α) (14). The coexistence of IVIG resistance and cholestatic hepatitis represents an exceptionally high-risk scenario for which optimal treatment strategies are poorly defined.

This report describes a 2-year-old child with IVIG-resistant KD complicated by cholestatic hepatitis and marked hypercytokinemia (TNF-α 89 pg/mL, IL-6 385 pg/mL). Despite failure of two IVIG doses and high-dose methylprednisolone pulse therapy, salvage infliximab (5 mg/kg) administered on illness day 9 achieved rapid defervescence and normalization of both inflammatory markers and liver function. Nevertheless, the patient progressed to giant CAAs (*Z*-score > 10) within two weeks. This case underscores a critical dilemma: suppression of systemic inflammation does not necessarily equate to coronary protection. We discuss the efficacy of infliximab in refractory KD with hepatic involvement, analyze potential reasons for CAA progression despite timely biologic intervention, and highlight the urgent need to redefine treatment success beyond fever control.

Because the data used in this study were retrieved from the hospital's electronic records and the research imposed no additional physical harm to the participants, the ethics committee waived the requirement for informed consent. The study was conducted in accordance with the Declaration of Helsinki.

## 2 Case presentation

A 2-year-old Han Chinese male child was admitted to Shaoxing Maternal and Child Health Hospital due to “fever for 1 day” (maximum temperature 38.1 °C). The patient was born prematurely but achieved normal developmental milestones. He had a history of respiratory syncytial virus infection 18 months prior, with no recurrence thereafter. No family history of genetic, hereditary, or autoimmune diseases was reported. Physical examination on admission revealed only mild fever, with no other obvious abnormalities, no rash, lymphadenopathy, or mucocutaneous changes. His height was 91.5 cm, weight 12 kg, heart rate 140 bpm, blood pressure 89/43 mmHg, and respiratory rate 34 breaths/min.

The child's condition progressed rapidly during hospitalization. On the 2nd day of hospitalization (illness day 3), a generalized maculopapular rash appeared, involving the trunk and limbs. On the 3rd day of hospitalization (illness day 4), other typical manifestations of KD appeared, including cervical lymphadenopathy (right cervical lymph node 1.5 cm × 1.0 cm, tender, non-adherent to surrounding tissues), erythema and chapping of the lips, strawberry tongue, and edema of the hands and feet. These clinical manifestations fully met the diagnostic criteria for typical KD (15).

**Figure 1:**
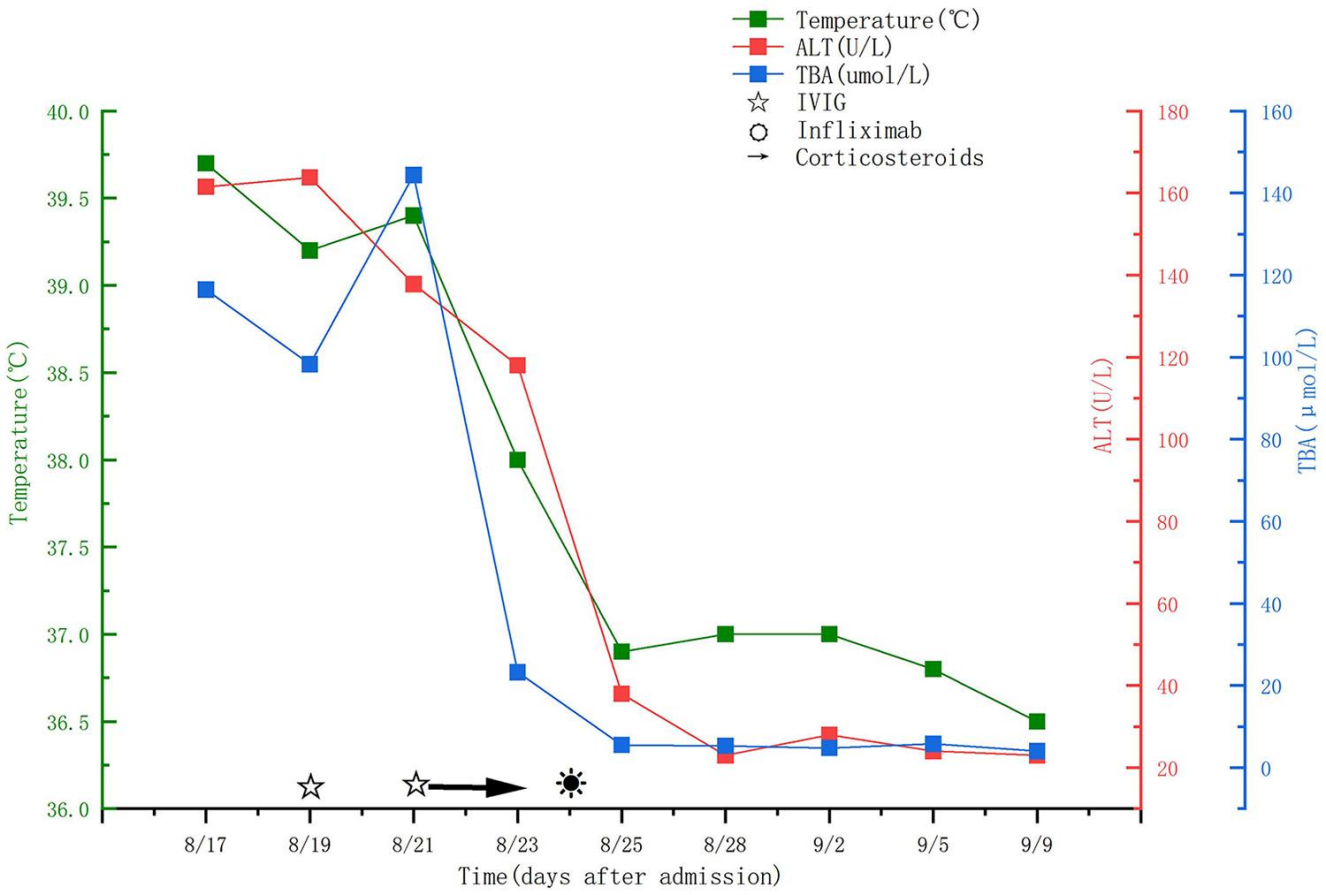
Temperature, ALT, TBA, and medication duration during hospitalization. This figure shows the dynamic changes of temperature, alanine aminotransferase (ALT), and total bile acids (TBA) during the child's hospitalization, as well as the administration time points of intravenous immunoglobulin (IVIG), methylprednisolone, and infliximab. It can be clearly seen that the child's fever subsided rapidly and liver function indicators gradually improved after infliximab administration.

Laboratory examinations on the 3rd day of hospitalization (4th day of illness) indicated severe systemic inflammation and cholestatic hepatitis (Figure 1). Hematological findings included white blood cell (WBC) count 28.23 × 10^9^/L (neutrophils 78.3%), hemoglobin 105 g/L, platelet count 356 × 10^9^/L. Inflammatory indicators: C-reactive protein (CRP) 162.31 mg/L (normal range <8 mg/L), erythrocyte sedimentation rate (ESR) 85 mm/h (normal range 0–10 mm/h). Liver function indicators: alanine aminotransferase (ALT) 161.53 U/L (normal range 7–40 U/L), aspartate aminotransferase (AST) 370.90 U/L (normal range 13–35 U/L), alkaline phosphatase (ALP) 324.62 U/L (normal range 45–125 U/L), gamma-glutamyl transferase (GGT) 85.52 U/L (normal range 7–32 U/L), total bilirubin 51.18 μmol/L (normal range 3.4–17.1 μmol/L), direct bilirubin 30.35 μmol/L (normal range 0–6.8 μmol/L), total bile acids (TBA) 116.44 μmol/L (normal range 0–10 μmol/L). Hypoalbuminemia was also present (albumin 27.06 g/L, normal range 35–55 g/L), further confirming the presence of systemic inflammation. Serum electrolytes on admission revealed hyponatremia (Na 124.9 mmol/L, reference 136–145), hypokalemia (K 3.39 mmol/L, reference 3.5–4.5), and normal chloride level (Cl 98.0 mmol/L, reference 98–107). Cytokine profiling showed significantly elevated levels of pro-inflammatory cytokines: interleukin-6: 385 pg/mL (normal range <7 pg/mL), interleukin-8: 196 pg/mL (normal range <25 pg/mL), interferon-gamma: 128 pg/mL (normal range <18 pg/mL), tumor necrosis factor-alpha: 89 pg/mL (normal range <8 pg/mL), while interferon-alpha levels were normal (12 pg/mL, normal range <15 pg/mL). Serological examinations for hepatitis A, B, C, Epstein–Barr virus, cytomegalovirus, and respiratory syncytial virus were all negative, ruling out infectious causes of liver injury. Abdominal ultrasound performed on the same day revealed a normal-sized liver with homogeneous echotexture and no space-occupying lesions; the gallbladder was normal in size without wall thickening, hydrops, biliary sludge, or stones; the common bile duct was not dilated; and the pancreas and spleen were unremarkable. These findings effectively excluded gallbladder hydrops, biliary obstruction, or alternative hepatobiliary pathology, supporting the attribution of cholestatic hepatitis to KD-associated systemic inflammation.

**Figure 2:**
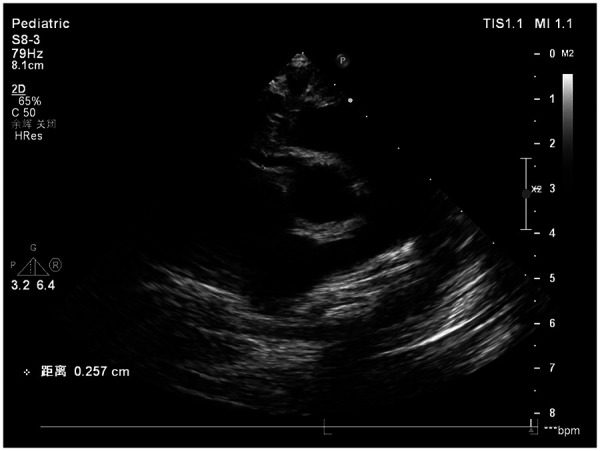
Echocardiography on the 4th day of hospitalization (right coronary artery *Z*-score 2.06). This echocardiogram shows mild dilation of the right coronary artery (*Z*-score 2.06) and no obvious abnormalities of the left coronary artery in the child on the 4th day of hospitalization (5th day of illness), suggesting early coronary artery involvement. *Z*-scores were calculated using the Canadian reference equations (16).

Echocardiography on hospital day 4 (illness day 5) demonstrated mild dilation of the right coronary artery (*Z*-score 2.06) (Figure 2), while the left coronary artery appeared normal. *Z*-scores were calculated using the Canadian reference equations (16).

First-line standard treatment was initiated on the 3rd day of hospitalization (4th day of illness): IVIG 2 g/kg (single infusion within 12 h) combined with clopidogrel 1 mg/kg/day (oral, once daily). Aspirin was avoided due to the presence of severe cholestatic hepatitis with marked elevation of liver enzymes and hypoalbuminemia (albumin 27.06 g/L), which increase the risk of Reye syndrome, hepatotoxicity, and bleeding complications from elevated free-drug concentrations. According to the 2017 AHA Kawasaki disease guideline (1), aspirin should be used with caution or avoided in patients with significant hepatic dysfunction; alternative antiplatelet agents such as clopidogrel are acceptable in such settings. Clopidogrel at 1 mg/kg/day has been demonstrated to be safe and effective in several studies of KD patients with aspirin contraindications (12). After the completion of IVIG infusion, the child still had persistent high fever (maximum temperature 39.8 °C), and clinical symptoms and laboratory indicators did not improve, indicating IVIG resistance.

Salvage therapy was adjusted on hospital day 5 (illness day 6): a second dose of IVIG 1 g/kg (8-h infusion) was administered in combination with high-dose methylprednisolone pulse therapy using a stepwise dose-escalation regimen: 13 mg/kg on day 1, followed by 20 mg/kg on days 2 and 3. This escalating regimen was chosen because the patient remained febrile after the initial 13 mg/kg dose and was diagnosed with refractory KD accompanied by extreme inflammation and early coronary dilation (right coronary artery *Z*-score 2.06). The reduced second IVIG dose (1 g/kg rather than the conventional 2 g/kg) was selected because the patient already had severe cholestatic hepatitis and hypoalbuminemia. According to our institutional protocol for IVIG-resistant KD with hepatic involvement, a 1 g/kg IVIG dose combined with corticosteroids provides adequate anti-inflammatory effect while minimizing hepatobiliary stress, an approach supported by published literature (10). The methylprednisolone step-dosing strategy (13 → 20 → 20 mg/kg) was employed to rapidly halt the progression of coronary vasculitis in this refractory setting, consistent with reports that high-dose methylprednisolone (15–20 mg/kg/day) can swiftly control inflammation and reduce coronary sequelae in refractory KD (17). However, this intensive regimen also failed to control the disease: fever persisted, and repeat inflammatory markers showed only a limited decrease (CRP: 158.76 mg/L, IL-6: 372 pg/mL, IL-8: 189 pg/mL), confirming the diagnosis of refractory KD. On hospital day 6 (illness day 7), two days prior to infliximab administration, the patient's CRP level was 58.81 mg/L.

From illness day 1 to day 8, the patient exhibited a persistent fever pattern with temperatures ranging from 38.0 °C to 40.0 °C. A transient decrease to 38.0 °C was observed on illness day 6 (hospital day 5, following the second IVIG and initial methylprednisolone dose), but fever recurred the next day (illness day 7, hospital day 6) with a temperature of 38.4 °C and persisted through illness day 8 (hospital day 7, 38.0 °C). This recurrent fever pattern confirmed ongoing disease activity rather than natural remission.

On the 8th day of hospitalization (9th day of illness), after multidisciplinary consultation and obtaining informed consent from the child's parents, salvage therapy with infliximab (5 mg/kg, single infusion within 2 h) was administered, and clopidogrel 1 mg/kg/day was continued. Notably, the child's fever completely subsided within 24 h after infliximab infusion, and clinical symptoms (rash, lip changes, hand and foot edema) gradually improved. Sustained normothermia (≤37.0 °C) was maintained throughout the remainder of hospitalization.

Serial laboratory testing demonstrated progressive improvement following infliximab therapy. On the 9th day of hospitalization (1 day after infliximab administration), CRP decreased to 31.27 mg/L. On the 10th day of hospitalization (2 days after infliximab), CRP further declined to 18.64 mg/L, ALT: 89.62 U/L, AST: 156.30 U/L, total bilirubin 32.15 μmol/L, TBA: 68.32 μmol/L. On the 14th day of hospitalization (6 days after infliximab), inflammatory indicators returned to normal (CRP: 7.8 mg/L, ESR: 9 mm/h), and liver function indicators improved significantly (ALT: 42.31 U/L, AST: 38.70 U/L, total bilirubin: 16.8 μmol/L, TBA: 9.76 μmol/L). Cytokine profiling showed that pro-inflammatory cytokine levels decreased significantly to near-normal ranges (interleukin-6: 12 pg/mL, interleukin-8: 32 pg/mL, interferon-gamma: 25 pg/mL, tumor necrosis factor-alpha: 10 pg/mL). No adverse reactions (such as infusion reactions, hypotension, allergic rash) occurred during infliximab treatment.

**Figure 3:**
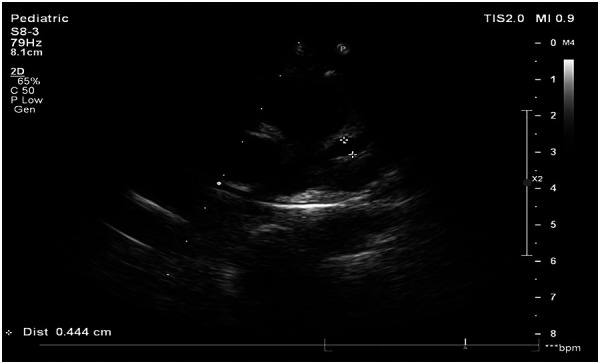
Echocardiography of the left anterior descending coronary artery on the 11th day of hospitalization (*Z*-score 8.46). *Z*-scores calculated as per Figure 2.

Despite effective control of systemic inflammation and significant recovery of liver function, serial echocardiographic follow-up revealed progressive coronary artery dilation. On hospital day 11 (illness day 12), echocardiography demonstrated significant dilation of the left anterior descending coronary artery (*Z*-score 8.46) (Figure 3).

**Figure 4:**
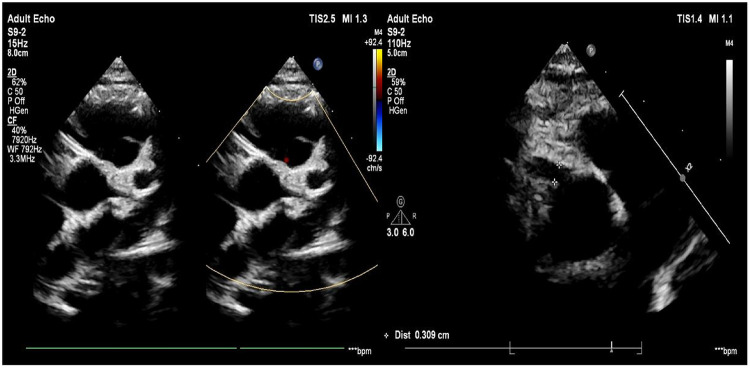
Echocardiography on the 24th day of hospitalization (left anterior descending coronary artery *Z*-score 10.51, right coronary artery *Z*-score 10.00). *Z*-scores calculated as per Figure 2.

On hospital day 24 (illness day 25), echocardiography indicated further progression to giant coronary artery aneurysms (CAAs), with a *Z*-score of 10.51 for the left anterior descending coronary artery and 10.00 for the right coronary artery (Figure 4), with the patient's CRP level had further normalized to 6.2 mg/L.

By illness day 20, the patient's CRP level had normalized to <0.2 mg/L. However, despite sustained biochemical remission, echocardiography on illness day 25 revealed progression to giant CAAs (LAD *Z*-score 10.51, RCA *Z*-score 10.00), indicating ongoing coronary artery injury even after systemic inflammation was controlled.

**Figure 5:**
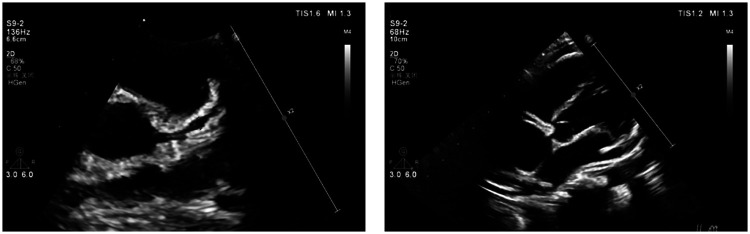
Echocardiography at six-month follow-up after discharge.

The child was discharged in stable condition on hospital day 28 (illness day 29). Long-term combined antiplatelet therapy (aspirin 4 mg/kg/day plus clopidogrel 1 mg/kg/day) was prescribed after discharge. Echocardiography performed six months after discharge demonstrated significant regression of coronary artery dimensions (Figure 5).

At the six-month follow-up visit after discharge, echocardiography was repeated to assess coronary artery remodeling. The examination revealed significant regression of the previously documented giant coronary artery aneurysms. The right coronary artery measured 0.20 cm at the origin and 0.23 cm at the distal segment; the left main coronary artery measured 0.25 cm; the left anterior descending artery measured 0.23 cm; and the left circumflex artery measured 0.16 cm (Figure 5). These findings indicate substantial improvement in coronary artery dimensions compared to the illness day 25 study (LAD *Z*-score 10.51, RCA *Z*-score 10.00), although mild residual dilation persists.

## 3 Discussion

This case describes a patient with IVIG-resistant KD complicated with cholestatic hepatitis. The clinical manifestations are consistent with the diagnostic criteria for typical KD, and after excluding infectious factors, cholestatic hepatitis is confirmed as a complication of KD, which is relatively rare in clinical practice.

The pathogenesis of cholestatic hepatitis complicated by KD remains incompletely clear. Previous studies have suggested that aggregation of NK cells in hepatic sinusoids during the acute phase of KD can lead to local inflammation and hepatocyte damage (18), while vasculitis of small hepatic and biliary vessels can affect bile flow, resulting in cholestasis (14). The significantly elevated TNF-α level in this child may further aggravate liver damage through immune-mediated mechanisms (14). Notably, infliximab treatment not only resolved systemic inflammation but also significantly improved liver function, suggesting that TNF-α may be a key mediator of cholestatic hepatitis complicated by KD, and targeted inhibition of TNF-α may be an effective strategy for the treatment of this rare complication.

IVIG resistance is a major risk factor for the development of CAAs in KD patients, and timely salvage therapy is crucial for improving prognosis. The child in this case did not respond to two doses of IVIG combined with high-dose methylprednisolone pulse therapy, indicating severe and refractory illness. Salvage therapy with infliximab achieved rapid fever resolution within 24 h, normalization of inflammatory indicators, and improvement of liver function, which is consistent with the efficacy of infliximab in IVIG-resistant KD confirmed by existing studies (12, 15, 17, 19). Since the first report of infliximab used in the treatment of refractory KD in 2004, this drug has been widely used in clinical practice, and multiple studies have confirmed that it can rapidly suppress inflammation and shorten the duration of fever (15, 17, 19). Its main mechanism of action is to specifically bind to TNF-α, inhibit its pro-inflammatory effects, thereby reducing vascular endothelial damage and suppressing the systemic inflammatory response (11, 20).

Of note, the patient's baseline TNF-α level was markedly elevated (89 pg/mL) and decreased to near-normal (10 pg/mL) after infliximab treatment. This rapid decline correlated temporally with fever resolution and improvement in liver function, supporting the central role of TNF-α in the pathogenesis of both KD vasculitis and KD-associated cholestatic hepatitis. However, despite effective neutralization of TNF-α, coronary artery damage continued to progress. This suggests that either the coronary vasculitis had already passed a “point of no return” before infliximab administration, or that other pro-inflammatory pathways (e.g., IL-1, IL-6) contributed independently to vascular injury. These observations underscore the need for earlier intervention and, potentially, combination biologic strategies in future trials.

Despite the excellent resolution of systemic inflammation and liver function following infliximab therapy, the patient still progressed to giant CAAs, a clinically troubling outcome that underscores the dissociation between fever control and coronary protection. This phenomenon is not unique to our case. In a large multicenter study, Hur et al. (13) explicitly reported that among IVIG-resistant KD patients who became afebrile after infliximab salvage therapy, 23.4% to 50% of subgroups still developed significant CAAs (*Z*-score > 5). Similarly, Burns et al. (15) observed that some patients exhibited coronary dilatation despite rapid defervescence after infliximab. These findings indicate that suppression of systemic inflammation does not equate to complete resolution of coronary arterial wall inflammation, and that defervescence and coronary protection may represent partially independent therapeutic end points.

Therefore, relying solely on fever resolution as a surrogate marker for treatment success may underestimate ongoing vascular injury. Several factors may explain the progression of CAAs in our patient: First, although infliximab was administered on illness day 9, which, in the context of refractory KD, represents a timely salvage intervention following failure of two IVIG doses and high-dose methylprednisolone, this timing still fell beyond the ideal window for primary coronary protection. Current evidence suggests that the optimal benefit of infliximab in preventing coronary artery damage may be achieved when given within the first 7–10 days of illness, ideally as part of intensified primary therapy rather than as rescue treatment (8, 13). This observation aligns with the concept that the severity of coronary artery injury is determined early in the disease course, before the peak of inflammation, when non-specific immune cells and cytokines cause irreversible damage to the vascular wall, as proposed by the protein homeostasis system hypothesis (6). Even if systemic inflammation is later controlled by agents like infliximab, the structural damage may already be established (7). Thus, defervescence and coronary protection may represent partially independent therapeutic end points. In our case, the patient received infliximab only after exhausting conventional second-line options, reflecting real-world step-up management rather than clinical delay. Nevertheless, this experience underscores that even “on-time” salvage therapy may be too late once severe coronary vasculitis has been triggered. Second, the patient had severe and persistent inflammation before infliximab administration (CRP up to 162.31 mg/L, TNF-α 89 pg/mL), which may have already caused irreversible damage to the coronary artery endothelium, leading to progressive aneurysm formation despite subsequent inflammation control. Third, individual differences in patient response to infliximab may exist, with some patients remaining at high risk of CAA even after effective inflammation control (4, 14).

The management of persistent CAAs after KD is another key clinical issue. Children with giant CAAs require long-term antiplatelet or anticoagulant therapy to prevent thrombosis and its complications (4). In this case, the patient was prescribed long-term combined antiplatelet therapy (aspirin + clopidogrel), which is a common clinical strategy for giant CAAs (4). For high-risk patients with giant or multiple CAAs, intensified anticoagulation therapy (such as adding low molecular weight heparin or dipyridamole) may be required, with individualized treatment based on aneurysm size, location, and patient condition (4). Regular echocardiographic follow-up is also essential to monitor aneurysm progression, stenosis, or thrombosis (3, 4).

This case also supports the proposal of integrating monoclonal antibody therapy into early intervention strategies for KD, rather than reserving it only for refractory cases. Early targeted biologic therapy (such as infliximab) can more effectively suppress the excessive inflammatory response in the acute phase, reduce vascular endothelial injury, and thereby reduce the risk of CAA formation. This concept is consistent with the recommendation for early, adequate immunomodulation (e.g., corticosteroids or IVIG) in hyperinflammatory syndromes, as emphasized in comparative analyses of KD and MIS-C (7). Additionally, cytokine profiling (such as TNF-α, IL-6 levels) may help identify patients at high risk of IVIG resistance and CAA, enabling personalized treatment decisions (14).

This case has certain limitations. First, as a single case report, the conclusions cannot be generalized to all patients with IVIG-resistant KD. Second, long-term follow-up of the child's coronary artery lesions and immune function was not performed, and the long-term efficacy and safety of infliximab still need further observation. Third, genetic markers associated with KD susceptibility or IVIG resistance were not detected, which may affect the analysis of individual treatment responses.

We also acknowledge that advanced imaging modalities, such as coronary CT angiography, cardiac MRI, or invasive coronary angiography, have not yet been performed due to the patient's age, the need for deep sedation/anesthesia, and parental refusal. This represents a significant limitation of the follow-up.

## 4 Conclusions

This case report demonstrates that infliximab can effectively suppress systemic inflammation and improve liver function in patients with IVIG-resistant KD complicated by cholestatic hepatitis. However, the development of giant CAAs despite successful inflammatory control underscores the critical importance of optimizing the timing and dosage of infliximab administration. Further randomized controlled trials are needed to establish definitive guidelines for the use of infliximab in IVIG-resistant KD regarding optimal timing, dosing, and patient selection. Incorporating cytokine profiling and advanced imaging modalities into clinical practice may help personalize treatment strategies, improve patient outcomes, and alleviate the long-term burden of cardiovascular complications.

## Data Availability

The original contributions presented in the study are included in the article/Supplementary Material, further inquiries can be directed to the corresponding author.

## References

[1] McCrindle BW Rowley AH Newburger JW Burns JC Bolger AF Gewitz M Diagnosis, treatment, and long-term management of kawasaki disease: a scientific statement for health professionals from the American Heart Association. Circulation. (2017) 135(17):e927–99. 10.1161/CIR.0000000000000484 28356445

[2] Newburger JW Takahashi M Burns JC. Kawasaki disease. J Am Coll Cardiol. (2016) 67(14):1738–49. 10.1016/j.jacc.2015.12.073 27056781

[3] Fukazawa R. Long-term prognosis of Kawasaki disease: increased cardiovascular risk? Curr Opin Pediatr. (2010) 22(5):587–92. 10.1097/MOP.0b013e32833e12f7 20717036

[4] Noval Rivas M Arditi M. Kawasaki disease: pathophysiology and insights from mouse models. Nat Rev Rheumatol. (2020) 16(7):391–405. 10.1038/s41584-020-0426-0 32457494 PMC7250272

[5] Agarwal S Agrawal DK. Kawasaki disease: etiopathogenesis and novel treatment strategies. Expert Rev Clin Immunol. (2017) 13(3):247–58. 10.1080/1744666X.2017.1232165 27590181 PMC5542821

[6] Lee KY Rhim JW Kang JH. Kawasaki disease: laboratory findings and an immunopathogenesis on the premise of a “protein homeostasis system”. Yonsei Med J. (2012) 53(2):262–75. 10.3349/ymj.2012.53.2.262 22318812 PMC3282974

[7] Rhim JW Kang JH Lee KY. Etiological and pathophysiological enigmas of severe coronavirus disease 2019, multisystem inflammatory syndrome in children, and Kawasaki disease. Clin Exp Pediatr. (2022) 65(4):153–66. 10.3345/cep.2021.01270 34809418 PMC8990954

[8] Nakamura Y Yashiro M Uehara R Sadakane A Tsuboi S Aoyama Y Epidemiologic features of Kawasaki disease in Japan: results of the 2009–2010 nationwide survey. J Epidemiol. (2012) 22(3):216–21. 10.2188/jea.JE20110126 22447211 PMC3798622

[9] Campbell AJ Burns JC. Adjunctive therapies for Kawasaki disease. J Infect. (2016) 72(Suppl):S1–5. 10.1016/j.jinf.2016.04.015 27241708

[10] Xue LJ Wu R Du G-L Xu Y Yuan K-Y Feng Z-C Effect and safety of TNF inhibitors in immunoglobulin-resistant Kawasaki disease: a meta-analysis. Clin Rev Allergy Immunol. (2017) 52(3):389–400. 10.1007/s12016-016-8581-4 27550227

[11] Brogan RJ Eleftheriou D Gnanapragasam J Klein NJ Brogan PA. Infliximab for the treatment of intravenous immunoglobulin resistant Kawasaki disease complicated by coronary artery aneurysms: a case report. Pediatr Rheumatol Online J. (2009) 7:3. 10.1186/1546-0096-7-3 19159441 PMC2646726

[12] Pan Y Fan Q Hu L. Treatment of immunoglobulin-resistant kawasaki disease: a Bayesian network meta-analysis of different regimens. Front Pediatr. (2023) 11:1149519. 10.3389/fped.2023.1149519 37520059 PMC10373588

[13] Hur G Song MS Sohn S Lee HD Kim GB Cho HJ Infliximab treatment for intravenous immunoglobulin-resistant Kawasaki disease: a multicenter study in Korea. Korean Circ J. (2019) 49(2):183–91. 10.4070/kcj.2018.0214 30468032 PMC6351283

[14] Hachiya A Kobayashi N Matsuzaki S Takeuchi Y Akazawa Y Shigemura T Analysis of biomarker serum levels in IVIG and infliximab refractory Kawasaki disease patients. Clin Rheumatol. (2018) 37(7):1937–43. 10.1007/s10067-017-3952-7 29302828

[15] Burns JC Best BM Mejias A Mahony L Fixler DE Jafri HS Infliximab treatment of intravenous immunoglobulin-resistant Kawasaki disease. J Pediatr. (2008) 153(6):833–8. 10.1016/j.jpeds.2008.06.011 18672254 PMC2856847

[16] Dallaire F Dahdah N. New equations and a critical appraisal of coronary artery Z scores in healthy children. J Am Soc Echocardiogr. (2011) 24(1):60–74. 10.1016/j.echo.2010.10.004 21074965

[17] Son MB Gauvreau K Burns JC Corinaldesi E Tremoulet AH Watson VE Infliximab for intravenous immunoglobulin resistance in Kawasaki disease: a retrospective study. J Pediatr. (2011) 158(4):644–649.e1. 10.1016/j.jpeds.2010.10.012 21129756

[18] Ohshio G Furukawa F Fujiwara H Hamashima Y. Hepatomegaly and splenomegaly in Kawasaki disease. Pediatr Pathol. (1985) 4(3-4):257–64. 10.3109/15513818509026899 3835550

[19] Song MS Lee SB Sohn S Oh JH Yoon KL Han JW Infliximab treatment for refractory Kawasaki disease in Korean children. Korean Circ J. (2010) 40(7):334–8. 10.4070/kcj.2010.40.7.334 20664742 PMC2910290

[20] Tremoulet AH Jain S Jaggi P Jimenez-Fernandez S Pancheri JM Sun X Infliximab for intensification of primary therapy for Kawasaki disease: a phase 3 randomised, double-blind, placebo-controlled trial. Lancet. (2014) 383(9930):1731–8. 10.1016/S0140-6736(13)62298-9 24572997

